# Reimagining Long-Term Services and Supports in a Post-Pandemic World

**DOI:** 10.1093/geroni/igab046.973

**Published:** 2021-12-17

**Authors:** Robert Applebaum, Katherine Abbott, Gretchen Alkema

**Affiliations:** 1 Miami University, Oxford, Ohio, United States; 2 The SCAN Foundation, Long Beach, California, United States

## Abstract

Prior to the global pandemic, the United States struggled to coordinate, deliver, and finance quality, person-centered long-term services and supports (LTSS) through the default primary payer, Medicaid. The pandemic highlights the challenges of not having a LTSS system. LTSS workers are underpaid, overworked, and turning over at alarming rates. Families face mounting pressures of caring for a growing number of loved ones, some with very complex care. Costs continue to climb, and quality indicators are not improving. While our approach to LTSS has improved, costs and quality challenges still dominate the landscape. We are at juncture when we need to reimagine the LTSS system, one that genuinely puts the care recipients and their caregivers at the heart of the system. The pandemic has provided some lessons about how to think differently about what long-term services can look like. Now is the time to embrace innovative opportunities building on this adversity.

